# Discrete Survival Time Constructions for Studying Marital Formation and Dissolution in Rural South Africa

**DOI:** 10.3389/fpsyg.2020.00154

**Published:** 2020-02-18

**Authors:** Jesca M. Batidzirai, Samuel O. M. Manda, Henry G. Mwambi, Frank Tanser

**Affiliations:** ^1^School of Mathematics, Statistics and Computer Science, University of KwaZulu-Natal, Pietermaritzburg, South Africa; ^2^Biostatistics Unit, South African Medical Research Council, Pretoria, South Africa; ^3^Department of Statistics, University of Pretoria, Pretoria, South Africa; ^4^Africa Health Research Institute, KwaZulu-Natal, South Africa; ^5^Lincoln Institute for Health, University of Lincoln, Lincoln, United Kingdom; ^6^School of Nursing and Public Health, University of KwaZulu-Natal, Durban, South Africa

**Keywords:** discrete time survival, multi-state models, multilevel models, competing risks, state transition

## Abstract

**Introduction:** Marriage formation and dissolution are important life-course events which impact psychological well-being and health of adults and children experiencing the events. Family studies have usually concentrated on analyzing single transitions including *Never Married* to *Married* and *Married* to *Divorced*. This does not allow understanding and interrogation of dynamics of these life changing events and their effects on individuals and their families. The objective of this study was to assess determinants associated with transitions between and within marital states in South Africa.

**Methods:** The population-based data available for this study consists of over 55, 000 subjects representing over 340, 000 person-years exposure from the Africa Health Research Institute (AHRI) in rural KwaZulu-Natal, South Africa. It was collected from 1 January 2004 to 31 December 2016. Multilevel multinomial, binary and competing risks regression models were used to model marital state occupation, transitions between marital states as well as investigate determinants of marital dissolution, respectively.

**Results:** Between the years 2006 and 2007, a subject was more likely to be married than never married when compared to years 2004 − 2005. After 2007, subjects were less likely to be married than never married and the trend reduced over the years up to 2016 [with *OR*=0.86, *CI*=(0.78; 0.94), *OR*=0.71, *CI*=(0.64; 0.78), *OR*=0.60, *CI*=(0.54; 0.67), *OR*=0.50, *CI*=(0.44; 0.56), and *OR* = 0.43, *CI* = (0.38; 0.48)] for periods 2008 − 2009, 2010 − 2011, 2012 − 2013, 2014 − 2015, and 2016, respectively. In 2008 − 2009, subjects were more likely to experience a marital dissolution than in the period 2004 − 2005 and the trend slightly reduces from 2010 until 2013 [*OR*=24.49, *CI*=(5.53; 108.37)]. Raising age at first sexual debut was found to be inversely associated with a marital dissolution [*OR* = 0.97;*CI* = (0.95; 0.99)]. Highly educated subjects were more likely to stay in one marital state than those who never went to school [*OR*=6.43, *CI*=(4.89; 8.47), *OR*=18.86, *CI*=(1.14; 53.31), and OR=2.96, CI=(1.96; 4.46) for being married, separated and widowed, respectively, among subjects with tertiary education]. As the age at first marriage increased, subjects became less likely to experience a marital separation [*OR* = 0.06, *CI* = (0.00; 1.11), *OR* = 0.05, *CI* = (0.00; 0.91), and *OR* = 0.04, *CI* = (0.00; 0.76) for subjects who entered a first marriage at ages 18 − 22, 23 − 29, and 30 − 40, respectively].

**Conclusion:** The study found that marrying at later ages is associated with a lower rate of marital dissolution while more educated subjects tend to stay longer in one marital state. Sexual debut at later ages was associated with a lower likelihood of experiencing a marital dissolution. There could, however, be some factors that are not accounted for in the model that may lead to heterogeneity in these dynamics in our model specification which are captured by the random effects in the model. Nonetheless, we may postulate that existing programs that encourage delay in onset of sexual activity for HIV risk reduction for example, may also have a positive impact on lowering rates of marital dissolution, thus ultimately improving psychological and physical health.

## 1. Introduction

Timing of marriage and marriage dissolution are associated with the psychological well-being and health of adults and children experiencing the events. Evidence suggests that early marriages and marital dissolutions increase the rates of stress, depression, high blood pressure, anxiety, aggression, suicide thoughts, and many other mental health disorders (Amato, 2005; Moon, 2011; Hashemi and Homayuni, 2017). Early marriages may also affect a woman's chance of educational and economic empowerment (Heward and Bunwaree, 1999; Amato, 2005; Hasselmo et al., 2015). On the other hand, one of the consequences of a marital dissolution where children are involved is child-headed families, which in turn adversely affects the development of children themselves. Children raised with divorced parents experience deferentially worse health and developmental profiles and lower survival rates compared to children living with stable and in union parents (Mackay, 2005). The rate of suicide (and suicidal thoughts) has been found to be associated with family dissolution, both for partners (Gove, 1973; Lillard and Panis, 1996; Kazan et al., 2016) and children (Kreitman, 1977; Gould et al., 1998). These health outcomes on vulnerable individuals could be due to stigma and societal norms that frown upon women (or men) who are divorced or separated and their children or due to thoughts of loss of material or financial belongings (Konstam et al., 2016).

While the above might be true, subjects may also get out of bad marriages so as to free themselves and have better well-beings. Despite these intertwining relationships and consequences, South Africans, including those in the rural areas, still find themselves in a system where they marry, separate, remarry, or become widowed while others remain in one marital state for a long period of time. For family planning practitioners and demographers to make informed decisions (for their intervention programs), they need to understand the patterns of movements or transitions between these marital states. Limited research on transitions between marital states has been done but in some instances, researchers would look at only transitions between two non-recurrent states, such as first marriage (see Bramlett and Mosher, 2001; Manda and Meyer, 2005; Hosegood et al., 2009) or divorce from marriage (Clark and Brauner-Otto, 2015). Robust statistical models have been developed and may be used to highlight issues on the dynamics in marital formation and dissolution. However, as Tanser et al. (2003) points out, a drawback of some statistically-driven models are that data sets used to develop the models are often of uncertain accuracy, models are not easily reproducible and the results are often applicable only to national or subregional scales.

We use multilevel discrete time to event models on a rich prospective data from a population-based cohort to study three marriage dynamics scenarios. We explore substantive issues concerning marital formation and dissolution in rural South Africa. Specifically, we investigate the determinants of marital state occupation, transition between marital states as well as marriage failure.

## 2. Methods

In this study, we will consider three scenarios: the marital state a subject occupies at given ages, transitions between marriage states and how certain states end. These are discussed below.

### 2.1. Marital State Occupation

At a given age, a subject is known to have a particular marital status. We are interested in determining which age groups are likely to occupy a particular marital state. However, subjects do not only stay in marital states without some contributing factors. For researchers to ascertain why a subject may be in a given marital state at a particular age group, they must first understand different marriage formation and dissolution dynamics. Therefore, it is important to study the factors leading to marital state occupation.

### 2.2. Single Transitions Between Marital States

The transition between marital states is of importance to understand. For instance, the age of entering a first-time marriage might be of interest to family planners and to the civilization of a society at large as age at first marriage is one example of a transition that is directly associated with a country's health, fertility and economic state (Manda and Meyer, 2005; Jones and Gubhaju, 2009). Furthermore, a widowed or separated subject may end up re-marrying (making a transition into a *Married* state again). For those who would have experienced a marital dissolution, it is important to determine what factors make them re-marry and which age groups tend to re-marry or remain in a marital dissolution state. Marriage counselors and other organizations seek to give advises to those who consider a re-marriage.

### 2.3. Termination of a Marriage State

Some marriage-supporting institutions, including religious communities, advocate for married subjects to stay married. However, due to life's unfortunate events, some marriages may fail. There are two main possible ways of marriage dissolution: separation and widowhood (death of a partner). As such, it is important to study how married subjects make a transition out of the *Married* state. Again, understanding these possible transitions out of a marriage would assist those organizations that seek to empower widowed women, for example.

### 2.4. Data

#### 2.4.1. Study Area

The data used in this study was collected by the Africa Health Research Institute (AHRI) which is situated in the rural area of northern KwaZulu-Natal of South Africa (Tanser et al., 2007). The surveillance area is near Mtubatuba, in the Umkanyakude rural district. It constitutes an area of 438 square kilometers with a highly mobile population of 90,000 people who are members of 11,000 households. These include all individuals reported by household informants as household members regardless of them being resident or non-resident (Tanser et al., 2007; Hosegood et al., 2009). In the event where a subject is not available to respond, the head of household would be the suitable alternative informant. The Africa Centre Demographic Information System (ACDIS) started data collection on 1 January 2004 and it is an ongoing study. However, for the purposes of this research, we only considered subjects who were followed up from January 2004 to December 2016. For all registered households and individuals, demographic and health information was collected every 4 months. Because the surveillance cohorts are dynamic, subjects may enter or leave the cohort through migration or births and deaths at any time (Tanser et al., 2007), thus we have a case of ragged study entries. The migration might be within the surveillance area itself (where subjects change households) or as a result of in-migration and out-migration, as defined by Dobra et al. (2017). As such, the participation rates at each wave is about 95% for household data collection. Therefore, to minimize non-response, where respondents are either non- resident or unavailable, suitable household members are selected as alternative informants.

#### 2.4.2. Data for This Study

Among other data sets, AHRI collects longitudinal data on life course history of marriage and marriage dissolution events. To best of our knowledge, this study is the first attempt to analyze these data with advanced multilevel discrete time to event methods to study dynamics in family formation and dissolution. Previous analyses have used different statistical methods, such as Hosegood et al. (2009) who used descriptive statistics to compare the 2000 and the 2006 cohorts. In the case where discrete time event history analysis methods were used it was on HIV (Tomita et al., 2017) or on different study cohorts with a different subject area (Clark et al., 2013; Houle et al., 2014; Clark and Brauner-Otto, 2015). Of the 69, 134 observations (across all ages), this study considered 59, 792 episodes from 56, 308 unique subjects (comprising 343, 758 person-years of follow up) who were aged between 17 and 65 years. These were enrolled between January 2004 and December 2016, with an average of 1.06 episodes per person. The subject who experienced the highest number of episodes in the data set had 8 episodes and subjects stayed in a state for an average of 3.07 years. From the available and usable data, four marital states were considered (*Never Married, Married, Separated*, and *Widowed*) and each subject would be in one of these states at each time of visit. Separation in this context refers to any marital dissolution other than death of a partner, which could be a legal divorce or an informal divorce, both permanent and temporary. *Married* state includes any type of marriage, such as traditional African marriage, polygamous marriage and civil marriage.

Subjects would move between the four marital states and the possible transition types to be considered for binary regression models are entry into first marriage (*Never Married* to *Married*), exiting a marriage (*Married* → *Separated* and *Married* → *Widowed*), and remarriage (*Separated* → *Married* and *Widowed* → *Married*). For the competing risks model, we considered only three marital states, *Married, Separated*, and *Widowed*. A married subject has two possible ways that the marriage can end: either through separation or death of a partner.

#### 2.4.3. Censoring

Due to the dynamic nature of the population, some subjects would not be available to respond to the interview at certain follow-ups and there might be no one in the household who could respond on their behalf. As a common phenomenon in longitudinal studies, this results in heavy censoring of the data. For subjects who have intermittent missing data, the last observation carried forward (LOCF) approach of data imputation was done. This method has been widely used in longitudinal studies as it assumes that the outcome remains constant at the last observed value after the dropout. For more and insightful details on the LOCF, we refer to Shao and Zhong (2003).

#### 2.4.4. Ethical Clearance

Ethical approval for the study conducted by AHRI was obtained from the University of KwaZulu-Natal's Ethics Committee (BE 169/15). Both informed verbal and written consent were sought from study participants and details regarding operational and methodological procedures of ACDIS are well-documented by Muhwava et al. (2008). In cases where a participant was under the age of 16, written consent was provided by their parent or guardian. However, in this study, we do not consider participants under the age of 16.

### 2.5. Statistical Analysis

For the analysis, we use age as the time scale and consider it as a discrete variable. The main advantage of discrete time analysis lies in its flexibility when modeling time-varying covariates We consider subjects between ages 17 and 65. Most cases in the African context for demography usually consider age for marriage to start at 15 years (Manda and Meyer, 2005). However, for this study, since there were no events occurring before 17 years of age, we opted to use a starting age of 17 years. It is then subdivided into *q* = 24 unit intervals between 17 and 65 years (which do not necessarily need to be equal). A subject's marital status is assumed to change not more than once within a period of 2 years, hence we chose to use 2-year time intervals in the analysis. Thus, we have intervals [17, 19], [19, 21], …[64, 65] which we denote as *t* = 1, 2, …, 24.

For assessing factors impacting the state occupied by a subject who possesses a number of characteristics at a particular age interval, a multinomial regression model is used (Grilli and Rampichini, 2007). For single transitions between marital states, into and out of a marriage, separate binary logistic regression models were utilized (Allison, 1982; Manda and Meyer, 2005; Steele, 2011; Clark et al., 2013). Separate response variables were created for the separate transition types, namely; marital dissolution (i.e., a dummy variable of 0 if subject is married and 1 if widowed or separated) or re-marriage (i.e., 0 if still widowed or separated and 1 if re-married). For analyzing ways of dissolving a marriage, a competing risks model (Allison, 1982; Jenkins, 1995; Steele, 2011) was used. We considered transitions out of a *Married* state, where *Separated* and *Widowed* are the competing events. A random variable, *Y*_*it*_, is created such that it is coded 0 whenever subject *i* is at risk of leaving a *Married* state, 1 when a transition is made into a *Separated* state and 2 when a transition is made into a *Widowed* state (the competing event).

Additionally, since observations are done on each individual repeatedly, there is possible correlation among observations within the same subject. There may exist other unobserved subject-specific factors which may influence marital state occupation or transition between marital states. For example, one person may have reasons for not remarrying after the death of a partner, while another may easily remarry shortly. It is, therefore, important to account for these variations by including a subject-specific random effect in the analysis. In the binary logistic regression models, there is a separate random effect for each type of transition and these are allowed to be normally distributed with a mean of 0 and a variance of σ^2^. Moreover, since the marital states are possibly recurrent in a subject, the competing risks regression model controls for this using a multilevel model. The correlations will enable us to understand how one subject who experienced one type of marital transition is likely to experience the other. Tomita et al. (2017) used the same data set on HIV, accounting for its multilevel nature. However, they used continuous-time survival methods. Using discrete- time metrics, we fitted these in STATA15.1 using its inbuilt standard tools **xtlogit** for random effects logit model and **gsem** for multinomial logit.

## 3. Results

### 3.1. Descriptive Statistics

The explanatory variables considered in this study include gender, if income is earned, if subject is employed, highest education level attained by subject, age at first sexual intercourse and age a first marriage for subjects who have ever been married. The median age at first sexual debut was 18 (mean = 18.03, min = 5, and max = 48) years for those who ever had sex. Table 1 shows the distribution of some variables of interest at entry into the study where applicable. In all categories of the variables, never married subjects had the highest proportion. At entry into the study, 13.6% of the female participants were married and 10.3% of the males were married. Most participants did not earn an income and of those who did, 24.4% were married and 69.5% were never married. Of those who did not earn an income, 5.9% were widowed.

**Table 1:** Distribution of explanatory variables for the 56,308 subjects aged between 17 and 65 years with marital status at entry to the study.

		**Marital status**			
**Variable**	**Total**	**Never married** ***N* (%)**	**Married** ***N* (%)**	**Separated** ***N* (%)**	**Widowed** ***N* (%)**
**EDUCATION**					
Nev went to Sch	657	258 (39.3)	193 (29.4)	5 (0.8)	201 (30.6)
Primary	4,408	3,510 (79.6)	621 (14.1)	7 (0.2)	270 (6.1)
High school	3,450	2,776 (80.5)	552 (16.0)	13 (0.4)	109 (3.2)
Tertiary	216	87 (40.3)	108 (50.0)	1 (0.5)	20 (9.3)
**GENDER**					
Female	32,360	25,319 (78.2)	4,407 (13.6)	92 (0.3)	2,542 (7.9)
Male	23,948	21.255 (88.8)	2,459 (10.3)	28 (0.1)	206 (0.9)
**INCOME IS EARNED**					
Yes	4,180	2,906 (69.5)	1,018 (24.4)	17 (0.4)	239 (5.7)
No	42,203	33,813 (80.1)	5.801 (13.7)	103 (0.2)	2.486 (5.9)
**IS EMPLOYED**					
Yes	14,644	11,567 (79.0)	2,487 (17.0)	52 (0.4)	538 (3.7)
No	39,972	33,565 (84.0)	4,174 (10.4)	63 (0.2)	2.170 (5.4)

Number of transitions (in person-years) into the different marital states are represented in Table 2, where most subjects remained in the *Never Married* state and the most common transition type was *Married* to *Widowed* with 1, 561 transitions followed by the *Never Married* to *Married* transition with 1, 274 transitions. During the follow-up period, most subjects remained in the *Never Married* state with 267, 284 person-years. The median age at first marriage was found to be 34 years. Of the 17, 124 who ever married, 29%, 38%, 28%, and 5% had their first marriage at ages ≤ 22, 23 − 30, 31 − 40, and ≥ 41 years groups, respectively.

**Table 2:** Number of transitions between marital states from 1 January 2004 to 31 December 2016 for the 56,308 subjects aged between 17 and 65 years.

**Previous marital status**	**Current marital status**				
	**Never married**	**Married**	**Separated**	**Widowed**	**Total**
Never Married	267,284	1,274	0	0	268,558
Married	0	53,970	75	1,561	55,606
Separated	0	55	711	0	766
Widowed	0	519	0	18,309	18,828
Total	267,284	55,818	786	19,870	343,758

**Figure 1:**
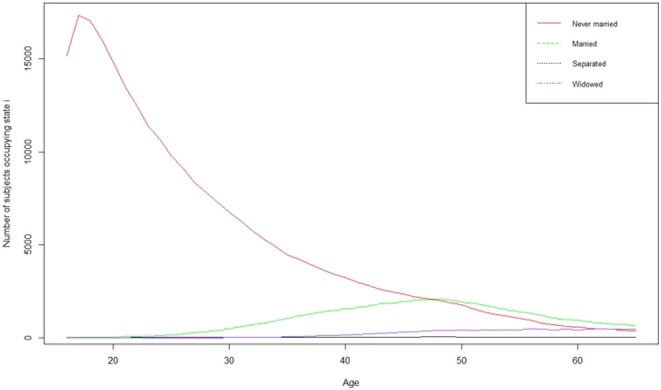
Distribution of subject in each state from age 17 to 65 years for the 267,284 person-years from 56,308 subjects in the study.

**Figure 2:**
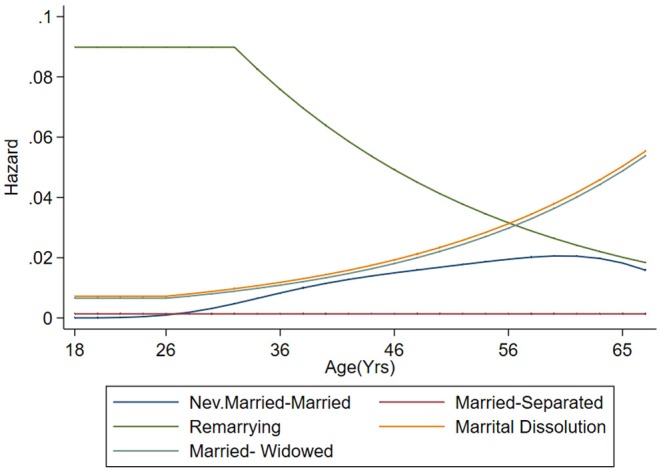
Baseline hazard for the different transitions for subjects aged 17–65 years for the 267,284 person-years from 56,308 subjects in the study.

**Figure 3:**
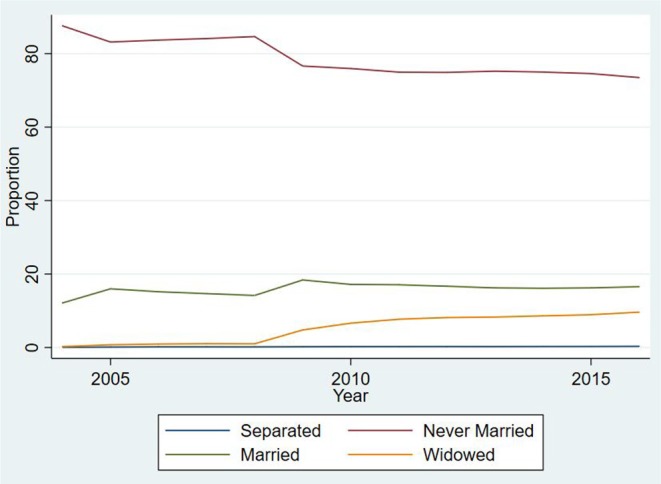
Proportions of various marital state occupations over time for subjects aged between 17 and 65 years from 2004 to 2016 for the 267, 284 person-years from 56,308 subjects in the study.

Figure 1 displays the proportion at each age, of subjects who were occupying each state. It is clear that below 46 years of age, the biggest part of the population was constituted by subjects who were never married. Moreover, as shown in Figure 2, the hazards of entering a first marriage were very low (close to 0) in the younger ages, but increased just slightly with age. Figure 3 also displays the trends over time for marital state occupation. Over the whole study period, the proportion of never married subjects was the highest. It decreased until 2008 and then stabilized afterwards. Proportion of married subjects was considerably low but slightly increased with time, then declined after 2009. The rates of marital separation were almost constant over time while those of widowhood started to increase slightly after 2008.

### 3.2. Results for Marital State Occupation

**Figure 4:**
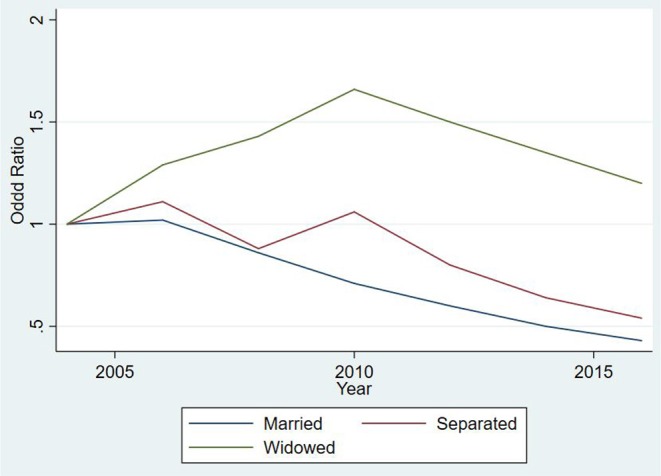
Odds Ratios for various marital state occupations over time when compared to never married subjects for subjects aged between 17 and 65 years.

We began by considering factors leading to staying in a marital state as shown in Table 3 below. The odds ratios are also displayed in Figure 4. All confidence intervals reported were at 95%. Compared to never married subjects, we assessed the odds of being married. Males had a significantly lower likelihood of being married than females [Odds Ratio (*OR*) for gender was 0.84(*CI* = 0.76; 0.93)]. Similarly, the odds ratio for primary, high school and tertiary education are 1.68(*CI* = 1.43; 1.98), 2.42(*CI* = 2.04; 2.86), and 6.43(*CI* = 4.89; 8.47), respectively, thus the likelihood of being married relative to never married were highest among subjects with tertiary education compared to those who never went to school. Those who did not earn income and those who were not employed had a lower likelihood of being in a *Married* state relative to *Never Married* [*OR* = 0.93(*CI* = 0.87; 1.01) and 0.93(*CI* = 0.86; 0.99), respectively]. Between the years 2006 and 2007, a subject was more likely to be married than never married when compared to years 2004 − 2005. After 2007, subjects were less likely to be married than never married and the trend reduced over the years up to 2016 [with *OR* = 0.86, *CI* = (0.78; 0.94), *OR* = 0.71, *CI* = (0.64; 0.78), *OR* = 0.60, *CI* = (0.54; 0.67), *OR* = 0.50, *CI* = (0.44; 0.56), and *OR* = 0.43, *CI* = (0.38; 0.48)] for periods 2008 − 2009, 2010 − 2011, 2012 − 2013, 2014 − 2015 and 2016, respectively. However, the variation in the likelihood of being married, between subjects was quite substantial [which was allowed to vary with a standard deviation of 0.22 (*CI*=0.21; 0.23)].

**Table 3:** Results for the marital state occupations for subjects aged between 17 and 65 years from 2004 − 2016 for the 56,308 subjects.

	**Married vs. N. married**	**Separated vs. N. married**	**Widowed vs. N. married**
**Covariates**	**Odds ratio (Conf. Int)**	**Odds ratio (Conf. Int)**	**Odds ratio (Conf. Int)**
**Constant**	0.01 (0.01; 0.01)^*^	0.00 (0.00; 0.00)^*^	0.00 (0.00; 0.00)^*^
**Age interval**	1.28 (1.27; 1.29)^*^	1.35 (1.27; 1.44)^*^	1.50 (1.46; 1.53)^*^
**Period** (Ref: 2004–2005)			
Period 2006–2007	1.02 (0.94; 1.10)	1.11 (0.51; 2.40)	1.29 (0.93; 1.80)
Period 2008–2009	0.86 (0.78; 0.94)^*^	0.88 (0.36; 2.16)	1.43 (1.02; 1.99)^*^
Period 2010–2011	0.71 (0.64; 0.78)^*^	1.06 (0.41; 2.80)	1.66 (1.18; 2.33)^*^
Period 2012–2013	0.60 (0.54; 0.67)^*^	0.80 (0.30; 2.09)	1.50 (1.06; 2.11)^*^
Period 2014–2015	0.50 (0.44; 0.56)^*^	0.64 (0.24; 1.68)	1.35 (0.95; 1.92)
Period 2016	0.43 (0.38; 0.48)^*^	0.54 (0.20; 1.43)	1.20 (0.84; 1.70)
**Gender** (Ref: Female)			
Male	0.84 (0.76; 0.93)^*^	0.42 (0.24; 0.73)^*^	0.09 (0.07; 0.11)^*^
**Income** (Ref: Yes)			
No	0.93 (0.87; 1.01)	0.96 (0.70; 1.32)	1.03 (0.92; 1.16)
**Is employed** (Ref: Yes)			
No	0.93 (0.86; 0.99)^*^	0.52 (0.35; 0.78)^*^	1.08 (0.97; 1.21)
**Highest education** (Ref: Never went to Sch)			
Primary	1.68 (1.43; 1.98)^*^	1.62 (0.59; 4.46)	1.34 (1.11; 1.62)^*^
High school	2.42 (2.04; 2.86)^*^	4.50 (1.52; 13.34)^*^	1.62 (1.29; 2.02)^*^
Tertiary	6.43 (4.89; 8.47)^*^	14.86 (4.14; 53.31)^*^	2.96 (1.96; 4.46)^*^
Age at first sex	1.00 (0.99; 1.01)	1.01 (0.98; 1.03)	1.00 (0.99; 1.01)
**σ_*i*_**	0.22 (0.21, 0.23)^*^	0.09 (0.08, 0.10)^*^	0.43 (0.23, 0. 63)^*^

* *Statistically significant variable*.

For *Separated* vs. *Never* *Married* state occupation, although there is heterogeneity among subjects (with a standard deviation of the heterogeneity component of 0.09(*CI* = 0.08; 0.10)), males and those without employment were less likely to be in a separated state than females and those who were employed [*OR* = 0.42(*CI* = 0.24; 0.73) and *OR* = 0.52(*CI* = 0.35; 0.78) for gender and employment, respectively]. When compared to those who never went to school, the more educated a subject became, the higher the likelihood of being in a *Separated* state [*OR* = 1.62(*CI* = 0.59; 4.46), 4.50(*CI* = 1.52; 13.34) and 14.86(*CI* = 4.14; 53.31) for primary, secondary, and tertiary education, respectively].

When comparing subjects who occupied a *Widowed* state relative to those who never married, males were less likely to be widowed compared to females [*OR* = 0.09(*CI* = 0.07; 0.11)]. When compared to subjects without any education, the likelihood of occupying a widowed state significantly increased with increasing level of education [*OR* = 1.34(*CI* = 1.11; 1.62), 1.62(*CI* = 1.29; 2.02), and 2.96(*CI* = 1.96; 4.46) for primary, secondary, and tertiary education, respectively]. There existed some significant subject-to-subject variation in the likelihood of the *Widowed* vs. *Never* *Married* state occupation [standard deviation of the heterogeneity component, *OR* = 0.431(*CI* = 0.231; 0.631)].

### 3.3. Results for Single Marital State Transitions

**Figure 5:**
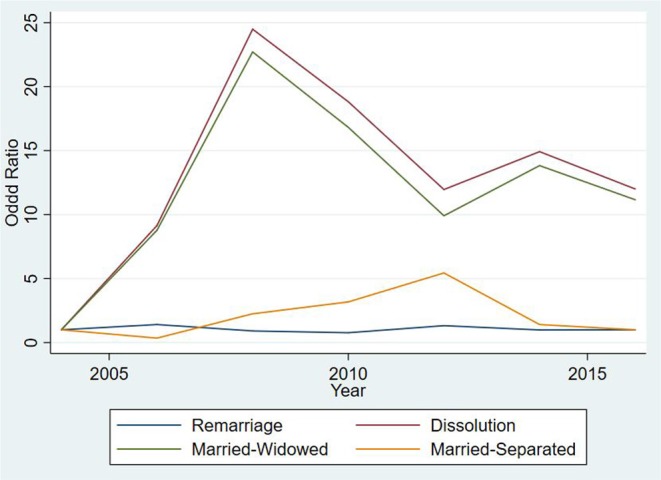
Odd ratios for transitions between marital states over time for subjects aged between 17 and 65 years.

Results of the binary transitions are displayed in Table 4 below. These transitions include marital dissolution, re-marrying after a dissolved marriage (*Separated* or *Widowed* → *Married*), marital dissolution due to a separation as well as a dissolution as a result of death of a partner. The hazards of transitions over the years are displayed in Figure 5. For each transition type, a separate baseline hazard was allowed. Using the smallest BIC, they all had a linear baseline effect of age except for the *married*→*separated* transition whose baseline was constant. These have been represented graphically in Figure 2. Additionally, there was homogeneity among subjects' re-marriage rates, since the standard deviation of the heterogeneity component was estimated as *SD* = 0. Increasing age significantly reduced the rate of re-marriage [*OR* = 0.92(0.88; 0.97)]. Males were significantly less likely to experience a marital dissolution than females [*OR* = 0.27, *CI* = (0.17; 0.45)]. Raising age at first sex was associated with a lower rate of marital dissolution [*OR* = 0.97(*CI* = 0.95; 0.99)]. The likelihood of having a marital dissolution was significantly associated with recent periods, with the period 2008 − 2009 having the highest effect [*OR* = 24;*CI* = (5.53 − 108.37)] [standard deviation of 1.44, *CI* = (1.04, 2.01)].

**Table 4:** Results for single transitions for the different family dynamics for subjects aged between 17 and 65 years between 2004 and 2016 for the 56,308 subjects.

	**Separated/Widowed** **→** **Married**		**Married** **→** **Separated/Widowed**		**Married** **→** **Widowed**		**Married** **→** **Separated**	
	**Odds ratio**	**Conf.Int**	**Odds ratio**	**Conf.Int**	**Odds ratio**	**Conf.Int**	**Odds ratio**	**Conf.Int**
**Constant**	0.37	(0.07; 2.06)	0.0	(0.00; 0.01)^*^	0.00	(0.00; 0.01)^*^	0.00	(0.00; 0.04)^*^
**Age interval**	0.92	(0.88; 0.97)^*^	1.05	(1.01; 1.09)^*^	1.06	(1.01; 1.10)^*^		
*Period* (Ref: 2004–2005)								
Period 2006–2007	1.41	(0.60; 3.29)	9.15	(2.04; 41.18)^*^	8.78	(1.93; 39.95)^*^	0.35	(0.01; 12.70)
Period 2008–2009	0.91	(0.44; 1.91)	24.49	(5.53; 108.37)^*^	22.71	(5.08; 101.59)^*^	2.25	(0.13; 38.46)
Period 2010–2011	0.77	(0.40; 1.51)	18.82	(4.22; 83.93)^*^	16.82	(3.72; 76.04)^*^	3.18	(0.24; 41.98)
Period 2012–2013	1.32	(0.68; 2.57)	11.96	(2.63; 54.33)^*^	9.91	(2.15; 45.65)^*^	5.44	(0.38; 78.70)
Period 2014–2015	0.99	(0.48; 2.02)	14.92	(3.26; 68.34)^*^	13.83	(2.98; 64.31)^*^	1.41	(0.07; 28.10)
Period 2016	1.00	(1.00; 1.00)	12.00	(2.53; 56.95)^*^	11.16	(2.31; 53.88)^*^	1.00	(1.00; 1.00)
**Gender** (Ref: Female)								
Male	1.16	(0.50; 2.67)	0.27	(0.17; 0.45)^*^	0.20	(0.12; 0.36)^*^	3.16	(0.66; 15.11)
**Income** (Ref: Yes)								
No	0.75	(0.46; 1.22)	0.80	(0.54; 1.17)	0.80	(0.53; 1.19)	0.98	(0.18; 5.17)
**Is employed** (Ref: Yes)								
No	1.14	(0.73; 1.76)	0.77	(0.56; 1.05)	0.81	(0.56; 1.12	0.35	(0.09; 1.34)
**Highest education** (Ref: Never went to school)								
Primary	0.81	(0.46; 1.43)	1.04	(0.61; 1.77)	0.97	(0.56; 1.68)	1.44	(0.66; 1.38)
High school	0.54	(0.29; 1.03)	0.86	(0.49; 1.52)	0.77	(0.43; 1.38)	2.71	(0.13; 54.45)
Tertiary	0.42	(0.13; 1.33)	0.52	(0.21; 1.28)	0.49	(0.19; 1.24)	0.00	(0.00, 0.00)
**Age at first sex**	1.02	(0.99; 1.05)	0.97	(0.95; 0.99)^*^	0.97	(0.95; 0.99)^*^	0.99	(0.91; 1.09)
**Age at first marriage** (Ref: Below 23 years)								
23–29	1.25	(0.81; 1.93)	1.06	(0.76; 1.50)	1.08	(0.75; 1.55)	0.55	(0.10; 2.94)
30–40	1.86	(1.15; 3.01)^*^	0.65	(0.44; 0.98)^*^	0.65	(0.43; 0.99)^*^	0.38	(0.06; 2.56)
41–65	0.92	(0.27; 3.19)	0.35	(0.16; 0.76)^*^	0.40	(0.18; 0.87)^*^	1.00	(1.00; 1.00)
**σ_*i*_**	0.00	0.00; 0.00	1.44	(1.04, 2.01)^*^	1.50	(1.07; 2.09)^*^	3.43	(2.50; 4.72)^*^

* *Statistically significant variable*.

Males were significantly less likely to experience a *Married* to *Widowed* transition, when compared to females [*OR* = 0.20, *CI* = (0.12; 0.36)]. The rest of the covariates did not significantly affect transition into widowhood. However, there existed some variation between subjects which is unaccounted for in the analysis [standard deviation of 1.50(*CI* = 1.07; 2.09)]. Lastly, higher ages at first marriage were associated with lower rates experiencing a *Married*→*Separated* transition for those who entered first marriages before the age of 40. The likelihood of a *Married*→*Separated* transition slightly decreased with increasing age at first marriage [*OR* = 0.55, *CI* = (0.10; 2.94) and *OR* = 0.38, *CI* = (0.06; 2.56) for age groups 23 − 29 and 30 − 40, respectively]. The standard deviation for random effects was 3.43(*CI* = 2.50; 4.72) which implies that there could be other factors associated with transitions from *Married* to *Separated* state which were not captured by the model that causes the variation.

### 3.4. Results for Termination of a Marriage

**Figure 6:**
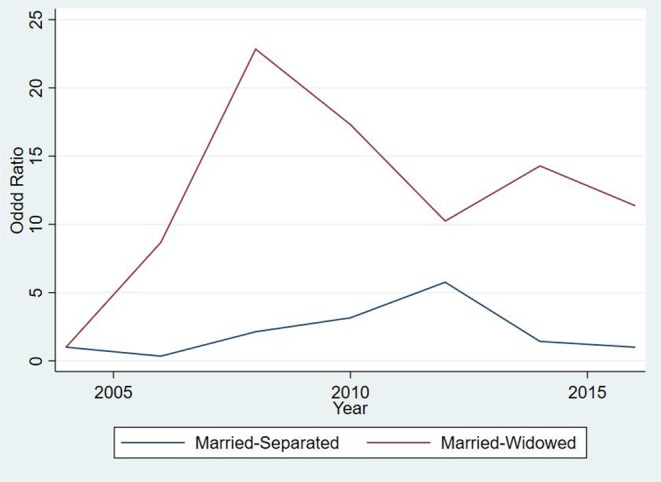
Odd ratios for dissolving a marriage over time for subjects between 17 and 65 years for the 267, 284 person-years from 56,308 subjects in the study.

Results of the competing risks model using the multivariate binary response are displayed in Table 5 below. The hazards of transitions over the years are displayed in Figure 6. These concern transitions out of a *Married* state, whose competing destination marital states were *Separated* and *Widowed*. The *Married*→*Separated* transition had a constant baseline age effect while the *Married*→*Widowed* had a linear age effect. Marrying at later ages was associated with a lower likelihood of transition from married to widowhood. [*OR* = 0.65, *CI* = (0.43; 0.99) and *OR* = 0.40, *CI* = (0.19; 0.88) for those who entered first marriage between 30 − 40 and ≥ 41 years, respectively]. Males were significantly less likely to experience a *Married*→*Widowed* transition when compared to women [*OR* = 0.20, *CI* = (0.12; 0.36)]. Substantial heterogeneity [OR of *SD* = 3.56(*CI* = 2.66, 4.77) and *SD* = 1.50(*CI* = 1.08, 2.09)] between subjects on the *Married*→*Separated* and *Married*→*Widowed* transitions, respectively, were observed.

**Table 5:** Results for exiting a marriage for subjects aged between 17 and 65 years since 2004–2016 from a population of 56,308 subjects.

	**Married** **→** **Separated**		**Married** **→** **Widowed**	
	**Odds ratio**	**Conf.Int**	**Odds ratio**	**Conf. Int**
**Constant**	0.00	(0.00; 0.03)^*^	0.00	(0.00; 0.00)^*^
**Age interval**			1.06	(1.01; 1.10)^*^
**Period** (Ref: Years 2004–2005)				
Period 2006–2007	0.34	(0.01; 13.00)	8.68	(1.91; 39.48)^*^
Period 2008–2009	2.13	(0.12; 37.46)	22.84	(5.10; 102.21)^*^
Period 2010–2011	3.15	(0.23; 42.422)	17.31	(3.82; 78.39)^*^
Period 2012–2013	5.76	(0.39; 85.39)	10.24	(2.22; 47.30)^*^
Period 2014–2015	1.42	(0.07; 29.14)	14.28	(3.07; 66.48)^*^
Period 2016	1.00	(1.00; 1.00)	11.38	(2.35; 55.02)^*^
**Gender** (Ref: Female)				
Male	3.47	(0.71; 17.04)	0.20	(0.12; 0.36)^*^
**Income** (Ref: Yes)				
No	0.98	(0.18; 5.32)	0.80	(0.54; 1.19)
**Is employed** (Ref: Yes)				
No	0.35	(0.09; 1.35)	0.81	(0.58; 1.13)
**Highest education** (Ref: Never went to sch)				
Primary	1.41	(0.06; 32.19)	0.97	(0.56; 1.67)
High school	2.71	(0.13; 57.09)	0.76	(0.43; 1.36)
Tertiary	1.00	(1.00; 1.00)	0.47	(0.19; 1.21)
**Age at first marriage** (Ref: Below 23)				
23–29	0.53	(0.10; 2.99)	1.08	(0.76; 1.54)
30–40	0.38	(0.05; 2.73)	0.65	(0.43; 0.99)^*^
41–65	1.00	(1.00; 1.00)	0.40	(0.19; 0.88)^*^
**Age at first sex**	1.00	(1.00; 1.00)	1.00	(1.00; 1.00)
**σ_*i*_**	3.56	(2.66; 4.77)^*^	1.50	(1.08; 2.09)^*^
**Cov_*ms* − *mw*_**	0.07			

* *Statistically significant variable*.

## 4. Discussion

Marriage and marriage dissolution are important life events that affect adults in a society. We used the rich data from one of Africa's largest population-based cohorts based in KwaZulu-Natal South Africa to model family formation and dissolution using a series of multilevel discrete time event models. In line with Garenne (2004) and Hosegood et al. (2009), our results demonstrate that marital rates are low (13.6% females and 10.3% males) and that age at first marriage is remarkably high in this population (median of 34 years). In addition, the results of the multilevel discrete time to event models revealed that marrying at later ages had a clear association with a low rate of marital dissolution, whilst more educated subjects and later age of sexual debut were associated with a lower likelihood of experiencing a marital dissolution. The role of these factors on marriage warrants further discussion and may be investigated in future research which can be supported by data.

The study has several strengths including the use of rich data from one of Africa's largest population-based cohorts and state-of-the-art statistical methods. However, even more advanced statistical models can still be used to model the transitions in this process simultaneously. As such, in addition to modeling the hazards and covariate effects, other methods may then be used to determine more useful statistics, such as expected waiting times, sojourn times, and transition probabilities which may then be used for prediction. Additionally, the random effects in this study were modeled using a normal distribution but a different distribution (such as gamma mixture, by Jenkins, 1997) may also be considered where different assumptions are made about the subjects. Different approaches may also be used to handle missing data.

Although marriages may get terminated as a result of poor (psychological) health (Wang and Amato, 2000), marital dissolution might lead to undesired health outcomes on both partners and children, such as stress, and high blood pressure. We found significant factors which contribute to a marital dissolution which may help with policy decision making. Many prevention programs in HIV encourage delay in sexual debut in order to reduce risk of HIV acquisition in the highly vulnerable youth (Karim et al., 2017). Thus, programs which encourage delay in sexual debut may have additional benefits in terms of reducing rates of marital dissolution thus ultimately improving psychological and physical health.

## Data Availability Statement

The datasets for this study can be found upon request from Africa Health Institute whose website is https://www.ahri.org/. Data sets with restricted access require a data access agreement to be completed. A request is then submitted to the applicable data custodian for specific data sets on the repository. For requests of *ad hoc* data sets, beyond the data sets archived on the Africa Centre data repository, these have to be directed to Africa Centre's Helpdesk (help@africacentre.ac.za).

## Ethics Statement

The studies involving human participants were reviewed and approved by Ethical approval for all the data collected by AHRI was obtained from the University of KwaZulu-Natal's Ethics Committee (BE 169/15). Written informed consent to participate in this study was provided by the participants' legal guardian/next of kin.

## Author Contributions

JB performed the literature review, data management, statistical analysis, and wrote the initial draft of the manuscript. SM conceived, designed, and developed the methodology of the study. HM helped with the design and methodology of the study. SM and HM reviewed the statistical analysis and helped with revision of the manuscript. FT contributed to the contextualization of the study findings and substantively helped with the revision of the manuscript. JB, SM, HM, and FT read and approved the final version of the manuscript.

### Conflict of Interest

The authors declare that the research was conducted in the absence of any commercial or financial relationships that could be construed as a potential conflict of interest.
